# Hand selection in a preferential reaching task: The effects of object location, orientation, and task intention in preadolescent children

**DOI:** 10.1002/brb3.1025

**Published:** 2018-08-11

**Authors:** Sara M. Scharoun Benson, Amanda Forsyth, Pamela J. Bryden

**Affiliations:** ^1^ Department of Kinesiology University of Windsor Windsor ON Canada; ^2^ Department of Kinesiology and Physical Education Wilfrid Laurier University Waterloo ON Canada

**Keywords:** children, hand selection, preferential reaching task, reaching

## Abstract

**Introduction and Methods:**

Hand selection was assessed in preadolescent children (ages 9–11) within a preferential reaching task to delineate the effects of object location, orientation, and task intention on the assessment procedure and compared to data previously acquired from young adults.

**Results:**

The observed differences support the notion that children are still in a process of refining their movements in attempt to discern the most efficient and effective patterns of behavior. Notwithstanding differences in performance, similarities between preadolescents and young adults also emerged. Greater right‐hand selection in right space and when the handle was oriented to the right indicate that object proximity and orientation influence efficiency and thus constrain hand selection in unimanual object manipulation and role‐differentiated bimanual manipulation.

**Conclusions:**

Together, findings add to our understanding of hand preference, unimanual and bimanual object manipulation.

## 1 INTRODUCTION

Handedness is traditionally assessed using measures of preference and performance. Preference indicates the hand typically selected for an action, whereas performance differentiates between abilities of the two hands when completing an action (McManus & Bryden, 1992). Questionnaires are used most frequently to confirm the direction (i.e., left or right) of hand preference, and a plethora of options are currently available (Scharoun & Bryden, 2014). The most commonly used questionnaires are the Annett Handedness Questionnaire (Annett, 1970a), Edinburgh Handedness Inventory (Oldfield, 1971), and Waterloo Handedness Questionnaire (Steenhuis, Bryden, Schwartz, & Lawson, 1990). Performance measures, in comparison, assess the degree (i.e., strength) of hand preference (Provins & Magliaro, 1993) through manual strength, speed, accuracy, and/or precision (Scharoun & Bryden, 2014). Peg‐moving tasks, such as the Annett Pegboard (Annett, 1970b) and Grooved Pegboard (Matthews & Klove, 1964), and manual aiming tasks (e.g., Roy & Elliott, 1986) are examples of performance assessments. It is generally understood that the preferred hand is more adept in performance, particularly for right‐handers (Annett, 1970b).

Beyond traditional measures, the assessment of manual midline crossing has also been used to quantify handedness. Failure to reach across the midline into contralateral space by the age of 3 or 4, which is part of the typical progression of sensorimotor development, may highlight a delay or problem that might manifest later in life (Michell & Wood, 1999). As such, findings that the development of hand preference influences reaching across the body into contralateral space (Carlier, Doyen, & Lamard, 2006; Doyen, Dufour, Caroff, Cherfouh, & Carlier, 2008) are supported by research with individuals with neurodevelopmental disorders (Gérard‐Desplanches et al., 2006; Groen, Yasin, Laws, Barry, & Bishop, 2008; Hill & Bishop, 1998).

One method of assessing manual midline crossing involves the assessment of hand selection for reaching throughout regions of hemispace. Bishop, Ross, Daniels, and Bright introduced the Quantification of Hand Preference task in 1996 as one method. Here, three playing cards were placed at 30‐degree intervals in hemispace, and participant hand selection was recorded in three tasks (card pointing, reaching, and posting). Findings revealed the ability to discriminate between left and right‐handers as a function of direction and degree of handedness (Bishop, Ross, Daniels, & Bright, 1996; Calvert, 1998; Doyen & Carlier, 2002), with high homogeneity and test–retest reliability (Doyen & Carlier, 2002). The card‐reaching task also proved to be sensitive to developmental processes. For example, Carlier et al. (2006) revealed significant differences between young children (ages 3–4) and older children (ages 9–10) and recorded fewer reaches into extreme regions of hemispace with the contralateral hand. Doyen et al. (2008) similarly demonstrated that adolescent children (ages 13–14) and adults reached across the body into contralateral space less often than preadolescent children (ages 7–12).

In addition to card‐reaching, researchers have asked participants to grasp and manipulate the same object in different movement contexts to discern how task complexity influences hand selection throughout regions of space. In one example, Bryden and Roy (2006) had right‐handed children (ages 3–10) reach for objects located at 45‐degree intervals in hemispace and perform simple (toss) and complex (place into receptacle of same size and shape) actions. More recent work from Bryden, Mayer, and Roy (2011) required right‐ and left‐handed children (ages 3–12) and young adults (ages 18–22) to pick‐up and use one of five objects (pencil, paintbrush, spoon, toothbrush, and toy hammer) and five identical dowels located in hemispace. Taken together, findings have revealed patterns of handedness concurrent with those that emerge from more traditional methods (e.g., questionnaires, peg‐moving tasks). As summarized in a review from Scharoun and Bryden (2014), young children (ages 3–5) are generally observed exploring the environment. As direction of hand preference is not yet established, the hand closest to the object is typically selected, reflecting a lack of differentiation between the two hands. Between the ages of 6 and 10, children have garnered more experience and have established which hand is more skilled; therefore, the preferred hand is selected, even in cases when it is not necessarily the most efficient. Finally, an “adult‐like” pattern of behavior is evident by approximately age 10 to 12, as preadolescent children learn to be less dependent on the preferred hand, and nonpreferred hand performance increases (Scharoun & Bryden, 2014).

This study aimed to further investigate the development of manual midline crossing in preadolescent children (ages 9–11) by extending Scharoun, Scanlan, and Bryden's (2016) work with young adults. In that study, a preferential reaching task with coffee mugs was used to assess hand selection. Mugs were placed in three regions of hemispace (right space, midline, and left space), and handle orientation also varied (toward, away, to the left, and to the right of the participant). Hand selection was assessed in four different tasks: (a) pick‐up; (b) pick‐up and pour; (c) pick‐up and pass; and (d) pick‐up, pour, and pass. For adults, a right‐hand preference emerged for unimanual (pick‐up; pick‐up and pass) tasks, especially when reaching for objects in right space. Bimanual tasks (pick‐up and pour; pick‐up, pour, and pass) revealed role differentiation between the two hands. The left hand was selected to reach for and stabilize the mug, leaving to right hand to mobilize the pitcher. Such findings were concurrent with the dynamic dominance hypothesis proposed by Sainburg et al. (Mutha, Haaland, & Sainburg, 2012; Przybyla, Good, & Sainburg, 2012; Sainburg, 2005; Sainburg & Kalakanis, 2000; Sainburg & Wang, 2002) and related literature assessing role‐differentiated bimanual manipulation (Babik & Michel, 2016; Kimmerle, Mick, & Michel, 1995; Ramsay & Weber, 1986). Here, the preferred hand is considered role‐differentiated bimanual manipulation hand preference, as it performs the more complex, mobilizing aspect of the task, whereas the nonpreferred hand serves a more subservient, stabilizing role (Guiard, 1987; Peters, 1994). In consideration of previous work comparing behaviors of adults and preadolescent children in unimanual object manipulation and role‐differentiated bimanual manipulation (e.g., Bryden & Roy, 2006; Bryden et al., 2011; Rudisch, Butler, Izadi, Birtles, & Green, 2018; Scharoun & Bryden, 2014), it was hypothesized that hand selection tendencies would differ from those of adults reported by Scharoun et al. (2016).

## 2 METHODS

### 2.1 Participants

Twenty‐four right‐handed children ages 9–11 (*n *= 6 age 9, 1 male, 5 female; *n *= 9 age 10, 6 male, 3 female; *n *= 9 age 11, 7 male, 2 female) participated in this research. Age was only recorded in years. Data were compared to 39 right‐handed undergraduate and graduate students (ages 18–30, exact ages not recorded; 14 male, 25 female) from Scharoun et al. (2016). Recruitment and testing procedures were reviewed and approved by the institution research ethics board. Informed consent was obtained from a parent/guardian of all participating children and adult participants. Child participants also provided verbal assent prior to participation.

### 2.2 Apparatus and procedures

Using the same methods as Scharoun et al. (2016), participants completed: (a) the 32‐item Waterloo Handedness Questionnaire (Steenhuis et al., 1990); and (b) a Preferential Reaching Task.

#### 2.2.1 Waterloo handedness questionnaire (WHQ)

A pen and paper task used to quantify hand preference, participants indicate their preferred hand for 32‐unimanual tasks by circling one of five responses: left always, left usually, both equally, right usually, and right always. A score of −2 (left always) to +2 (right always) is assigned to each response, and a total handedness score is computed (−64 to +64). A negative score is expected for left‐handed participants, and a positive score is expected for right‐handed participants.

#### 2.2.2 Preferential reaching task

**Figure 1:**
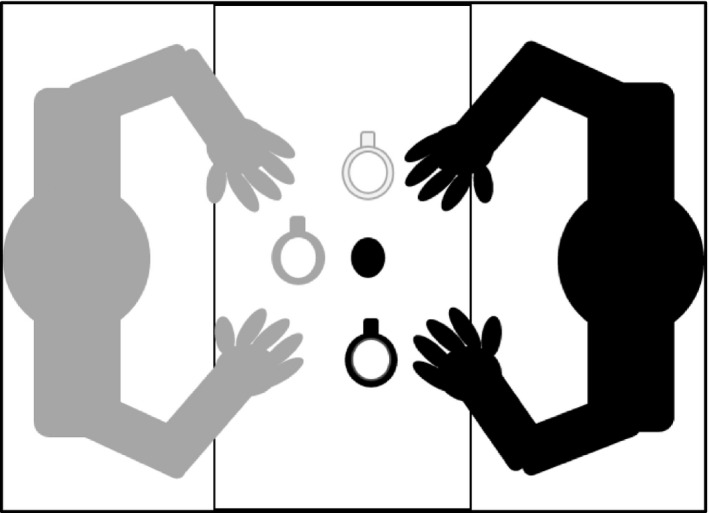
Study setup. The participant (black) sat directly across from the researcher (grey). Mugs were placed within reaching distance in left‐ and right space and at the midline. A water pitcher was placed at the participant's midline. Handles were oriented to the right, left, toward, or away from the participant

Participants were seated across from a researcher throughout the duration of the task. Three differently colored, yet identically proportioned coffee mugs were placed within reaching distance (20 cm) in left space (0°), at the midline (90°), and in right space (180°; Figure 1). The mug handle was oriented toward, away from, to the left or right of the participant. A water pitcher, without a handle, was placed at the participant's midline. The pitcher was filled with lukewarm water to simulate a warm beverage without the potential risk of burning that the use of boiling water would have introduced.

Participants were asked to complete four different tasks: (a) pick‐up (i.e., pick‐up the mug); (b) pour (i.e., pick‐up the mug and pour a glass of water); (c) pass (i.e., pick‐up the mug and pass it to the researcher; and (d) pour and pass (i.e., pick‐up the mug, pour a glass of water, and pass it to the researcher). Each trial started with the participants’ hands in a neutral position. Participants were not instructed how to complete the task (i.e., were not instructed which hand to use, or to grasp the mug by than handle); therefore, hand and grasp selection were freely selected. Each location–handle orientation–task combination was performed twice, for a total of 96 trials. Although trials were blocked by handle orientation, the order of task presentation was randomized. Participant behavior was recorded using a video camera placed in a front view. Hand selection to pick‐up the mug was coded offline, and the percentage of right‐hand selection was computed.

### 2.3 Data analysis

The percentage of right‐hand selection was the dependent measure. For analysis purposes, tasks were separated into unimanual (pick‐up, pass) and bimanual (pour, pour and pass). Using SPSS© statistics 24 software (http://scicrunch.org/resolver/SCR_002865), data were submitted to a 2 (group) × 2 (task) × 3 (location) × 4 (handle) mixed analysis of variance test with repeated measures.

## 3 RESULTS

Only significant results with large effect sizes ($\eta_{p}^{2}$ ≥ 0.14; Lakens, 2013) will be discussed in detail. All other nonsignificant effects and significant effects with small and medium effect sizes can be found in Table 1.

**Table 1:** Interactions that emerged with small and medium effect sizes

Effect	*F*‐statement
Unimanual	
Location × Group	*F* (2, 122) * *=* *3.097, *p *=* *0.049, $\eta_{p}^{2}$ * *=* *0.048
Task × Handle × Group	*F* (6, 366) * *=* *6.219, *p *<* *0.001, $\eta_{p}^{2}$ * *=* *0.093
Task × Location × Group	*F* (2, 122) * *=* *9.099, *p *<* *0.001, $\eta_{p}^{2}$ * *=* *0.130
Task × Location × Handle	*F* (6, 366) * *=* *4.717, *p *<* *0.001, $\eta_{p}^{2}$ * *=* *0.072
Task × Location × Handle × Group	*F* (6, 366) * *=* *3.041, *p *=* *0.034, $\eta_{p}^{2}$ * *=* *0.047
Bimanual	
Task × Group	*F* (1, 61) * *=* *4.581, *p *=* *0.036, $\eta_{p}^{2}$ * *=* *0.070
Location × Handle	*F* (6, 366) * *=* *5.186, *p *<* *0.001, $\eta_{p}^{2}$ * *=* *0.078
Location × Handle × Group	*F* (6, 366) * *=* *3.667, *p *=* *0.002, $\eta_{p}^{2}$ * *=* *0.057
Task × Location × Handle × Group	*F* (6, 366) * *=* *3.126, *p *=* *0.013, $\eta_{p}^{2}$ * *=* *0.049

### 3.1 Unimanual tasks (pick‐up, pass)

**Figure 2:**
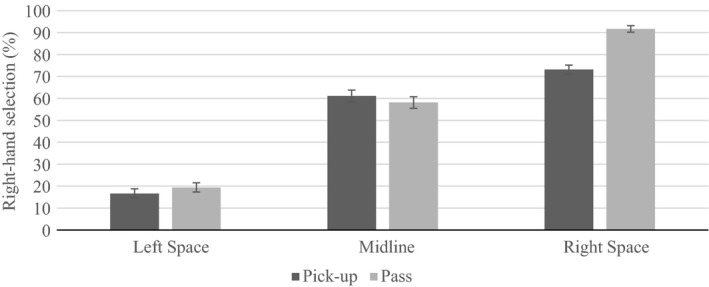
Right‐hand selection in right space was greater in pass compared to pick‐up

A main effect of handle (*F* (3, 183), *p *<* *0.001, $\eta_{p}^{2}$ = 0.166) revealed the right hand was selected more often when the handle faced right (*M *=* *61.51, *SD *=* *42.08) compared to all other orientations (left: *M *=* *49.47, *SD *=* *44.00, toward: *M *=* *52.25, *SD *=* *44.47, away: *M *=* *50.27, *SD *=* *45.78). Main effects of task (*F* (1, 61) * *=* *24.928, *p *<* *0.001, $\eta_{p}^{2}$ =0.290) and location (*F* (2, 122) * *=* *229.288, *p *<* *0.001, $\eta_{p}^{2}$ **=* *0.790) will be discussed within the significant two‐way interaction (*F* (2, 122) * *=* *28.615, *p *<* *0.001, $\eta_{p}^{2}$ **=* *0.319; Figure 2). Here, right‐hand selection in both tasks was most prevalent in right space, and least prevalent in left space compared to the midline. Furthermore, right hand selection was greater in right space for pass compared to pick‐up.

**Figure 3:**
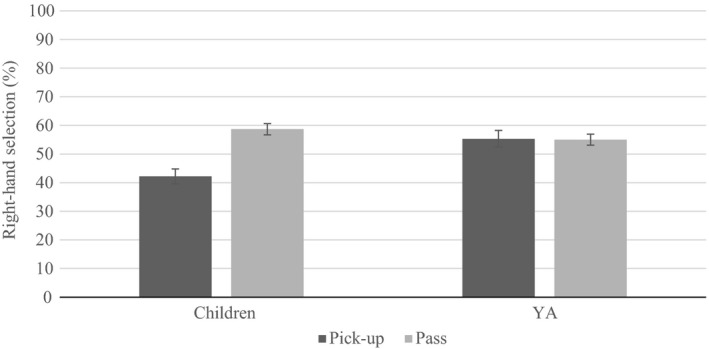
Children displayed less right‐hand selection in pick‐up compared to adults and compared to pass

**Figure 4:**
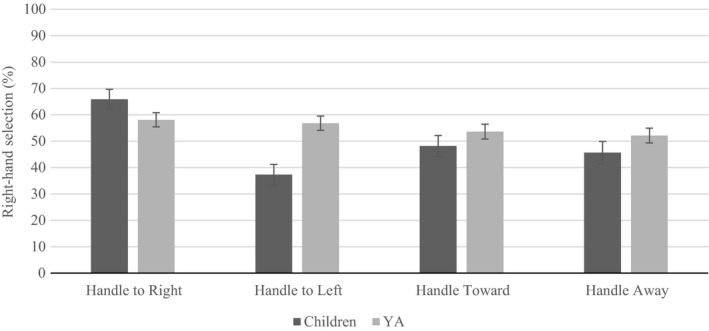
Unlike adults, differences in right‐hand selection emerged for children because of handle orientation. Furthermore, children displayed less right‐hand selection than adults when the handle faced left

Two‐way interactions between task and group (*F* (1, 61)* *= 26.943, *p* < 0.001, $\eta_{p}^{2}$ **=* *0.306; Figure 3), and handle and group (*F* (3, 183) * *=* *8.814, *p* < 0.001, $\eta_{p}^{2}$ **=* *0.126; Figure 4) were revealed. Significantly fewer right‐hand selections were displayed by children in pick‐up compared to pass and compared to adults. No difference emerged between children and adults in pass. Although no differences emerged in adults as a function of handle, children displayed an increase in right‐hand selection when the handle faced right, and the least when the handle faced. The proportion of right‐hand selection differed in all handle orientations, for children, except and away. Furthermore, when the handle faced left, adults displayed significantly more right‐hand selection than children.

### 3.2 Bimanual tasks (pour, pour and pass)

A main effect of location (*F* (2, 122) * *=* *41.942, *p *<* *0.001, $\eta_{p}^{2}$ **=* *0.407) revealed differences in all locations, with more right‐hand selection in right space (*M *=* *39.98, *SD *=* *46.38), compared to the midline (*M *=* *16.23, *SD *=* *34.10) and left space (*M *=* *11.31, *SD *=* *29.26). Furthermore, the main effect of handle (*F* (3, 183) * *=* *11.055, *p *<* *0.001, $\eta_{p}^{2}$ **=* *0.153) revealed more right‐hand selection when the handle was oriented to the right (*M *=* *27.38, *SD *=* *41.81), compared to left (*M *=* *16.80, *SD *=* *34.58), toward (*M *=* *20.05, *SD *=* *38.04), and away (*M *=* *21.43, *SD *=* *38.93).

## 4 DISCUSSION

In line with our hypothesis, differences emerged between the two groups. In particular, right‐hand selection differed as a function of location for adults in both tasks; however, for children, this was only the case for the pass task. In pick‐up, right‐hand selection at the midline and in right space did not differ. Findings support the notion that preadolescent children are still in a process of refining their movements in attempt to discern the most efficient and effective patterns of behavior. For example, Mason, Bruyn, and Lazarus (2013) stated, about unimanual and bimanual object manipulation, that “the transition time between the ages of 7–10 may therefore be used as a testing period to determine the most effective strategies for accomplishing a variety of task goals” (p. 162). Likewise, Hausmann, Waldie, & Corballis (2003) have described the transition from immature to mature motor control to persist through the ages 10–12. Findings revealed in the present study are thus likely attributed to this transition period. Here, hand selection was similar for the most part (i.e., in most trials); however, minute differences prevent the conclusion that adult‐like behavior is evident in this age group.

Role‐differentiated bimanual manipulation emerges early in a child's life, such that partially differentiates roles for each hand are displayed by approximately 13 months (Babik & Michel, 2016). Throughout development, the preferred hand establishes itself in a holding and stabilizing role, whereas the nonpreferred hand becomes more proficient at object manipulation (Kimmerle, Ferre, Kotwica, & Michel, 2010). Findings are concurrent with recent work from Rudisch et al. (2018). Here, 5‐ to 16‐year‐olds opened the lid of a transparent box with one hand and used the other hand to press a button inside the box. The leading hand altered between preferred and nonpreferred hand in two task conditions. Although no differences in task performance were revealed based on the leading hand, young children (ages 5–6) were more variable in temporal cooperation compared to older children (ages 7–9) and adolescents (ages 10–16). Additional analyses, which also assessed unimanual subtasks (i.e., opening the lid, pressing a button), revealed a decrease in smoothness of movement across all participants in bimanual actions. The difference was particularly large for young children, and more apparent between ages 5–6 and 7–9. The decrease in variability and increase in smoothness was interpreted as evidence of automatization (Cohen & Sternad, 2009) with increasing age (Rudisch et al. (2018). With respect to findings of the current study, it can be argued that children are in the process of automatization, albeit not yet to the same extent as would be expected in young adults.

Beyond developmental effects, a general tendency for greater right‐hand selection in right space and when the handle was oriented to the right was revealed in both unimanual and bimanual tasks. Extending the work of Scharoun et al. (2016) to include preadolescent children, findings are concurrent with the *kinaesthetic hypothesis* (Gabbard & Rabb, 2000), which states that preferred hand use will be limited in contralateral space, due to biomechanical constraints. As such, object proximity and efficiency constrain hand selection in preferential reaching task.

Findings also support the notion that an object's orientation influences efficiency and thus constrains hand selection (Scharoun et al., 2016). Previous work has demonstrated that when a handled mug is viewed, the typical response involves grasping the handle (Ellis & Tucker, 2000; Tucker & Ellis, 1998). That said, it is important to acknowledge participants in the present study were not explicitly instructed to grasp the mug by the handle and were thus free to grasp the mug as they best saw fit. Lindemann, Stenneken, Van Schie, and Bekkering (2006) argued that the action of grasping a handled mug only occurs when there exists an intention for mug use. More recent work from van Elk, van Schie, and Bekkering (2014a,2014b) has proposed that the human capacity to use tools and objects involves “automatic effects of affordances as well as context‐ and intentionally driven effects” (van Elk et al., 2014a; p. 240). Scharoun et al. (2016) assessed the propensity to grasp the mug by the handle, in addition to hand selection. They found that the handle was grasped more often in independent object manipulation. It can thus be argued that the hand selection tendencies, like grasp selection, are influenced by a multitude of different factors, including affordances, and the circumstances in which the action is performed. Together, findings are in line with previous assessments of preferential reaching (e.g., Bryden & Huszczynski, 2011), which have discussed the influence of object orientation and location, arm position and task complexity on hand selection.

Although we opted to separate unimanual and bimanual tasks for ease of analysis, as evident in results, and in line with previous reports, right‐hand selection was more prominent in unimanual tasks. Results offer support for role differentiation between the two hands in bimanual tasks. Concurrent with the dynamic dominance hypothesis proposed by Sainburg et al. (Mutha et al., 2012; Przybyla et al., 2012; Sainburg, 2005; Sainburg & Kalakanis, 2000; Sainburg & Wang, 2002) and the asymmetric division of labor hypothesis proposed by Guiard (1987), the left hand was selected to reach for and stabilize the mug, leaving to right hand to mobilize the pitcher. Findings from the two bimanual tasks (pour, pour and pass) revealed hand selection tendencies to be more similar than different when comparing preadolescent children to you adults. Although interactions involving the “group” factor did emerge, effect sizes were not large and therefore were not elaborated upon (see Table 1).

Taken together, findings revealed differences between hand selection patterns of preadolescent children and young adults. Here, it can be argued that children are still in a process of refining their movements in attempt to discern the most efficient and effective patterns of behavior. As such, unlike young adults, automatization of bimanual control is not yet established. Notwithstanding differences in performance, similarities between preadolescents and young adults also emerged. Greater right‐hand selection in right space and when the handle was oriented to the right provides support for the kinaesthetic hypothesis (Gabbard & Rabb, 2000). As such, object proximity and orientation influence efficiency and thus constrain hand selection in unimanual object manipulation and role‐differentiated bimanual manipulation.

## CONFLICT OF INTEREST

None declared.
